# Novel Active Food Packaging Films Based on Gelatin-Sodium Alginate Containing Beetroot Peel Extract

**DOI:** 10.3390/antiox11112095

**Published:** 2022-10-24

**Authors:** Moufida Chaari, Khaoula Elhadef, Sarra Akermi, Boutheina Ben Akacha, Mariam Fourati, Ahlem Chakchouk Mtibaa, Monia Ennouri, Tanmay Sarkar, Mohammad Ali Shariati, Maksim Rebezov, Slim Abdelkafi, Lotfi Mellouli, Slim Smaoui

**Affiliations:** 1 Laboratory of Microbial Biotechnology and Engineering Enzymes (LMBEE), Center of Biotechnology of Sfax (CBS), University of Sfax, Sfax 3018, Tunisia; 2 Laboratory of Biotechnology and Plant Improvement, Center of Biotechnology of Sfax, Sfax 3018, Tunisia; 3 Olive Tree Institute, University of Sfax, Sfax 3018, Tunisia; 4 Valuation, Security and Food Analysis Laboratory, National School of Engineers of Sfax, University of Sfax, Sfax 3038, Tunisia; 5 Department of Food Processing Technology, Malda Polytechnic, Bengal State Council of Technical Education, Government of West Bengal, Malda 732102, West Bengal, India; 6 Department of Scientific Research, Russian State Agrarian University—Moscow Timiryazev Agricultural Academy, 127550 Moscow, Russia; 7 Department of Scientific Research, V. M. Gorbatov Federal Research, Center for Food Systems, 26 Talalikhin St., 109316 Moscow, Russia; 8 Laboratory of Enzymatic Engineering and Microbiology, Algae Biotechnology Unit, Biological Engineering Department, National School of Engineers of Sfax, University of Sfax, Sfax 3038, Tunisia

**Keywords:** beetroot extract, active packaging, mechanico-physical and optical characteristics, minced beef meat, microbial spoilage, oxidative stability, shelf-life extension, multivariate analysis

## Abstract

Currently, the exploration of natural colorants from vegetal waste has gained particular attention. Furthermore, incorporation of these natural sources into biopolymers is an encouraging environmentally friendly approach to establishing active films with biological activities for food packaging. The present study developed bioactive antioxidant films based on gelatin-sodium alginate (NaAlg) incorporated with aqueous beetroot peel extract (BPE). Firstly, the effects of combining gelatin-NaAlg and BPE at 0.25, 0.5, and 1% on the mechanical, physical, antioxidant, and antibacterial properties of the films were analyzed. With increasing BPE, mechanico-physical properties and antioxidant and anti-foodborne pathogen capacities were enhanced. Likewise, when added to gelatin-NaAlg films, BPE remarkably increased the instrumental color properties. Moreover, during 14 days of storage at 4 °C, the impact of gelatin-NaAlg coating impregnated with BPE on microbial and chemical oxidation and on the sensory characteristics of beef meat samples was periodically assessed. Interestingly, by the end of the storage, BPE at 1% limited the microbial deterioration, enhanced the instrumental color, delayed chemical oxidation, and improved sensory traits. By practicing chemometrics tools (principal component analysis and heat maps), all data provided valuable information for categorizing all samples regarding microbiological and oxidative properties, sensory features, and instrumental color. Our findings revealed the ability of gelatin-NaAlg with BPE as an antioxidant to be employed as food packaging for meat preservation.

## 1. Introduction

Recently, the main concern of the food packaging field was the elaboration of functional and biodegradable materials that can completely substitute the derived forms of non-renewable petroleum-based sources [1,2,3]. Approximately 60% of plastics were estimated to be discarded in the environment without any appropriate treatment, which will last for centuries with very slow decomposing [4,5]. In response to this global environmental challenge, biodegradable films were developed. In addition, to serve consumer demands regarding healthy and safe food products, researchers and food packaging industries attend to develop natural, active, and environmentally friendly films [6,7]. In this regard, the biopolymers gained from renewable resources presently constitute a supportable alternative to petroleum-derived polymers, and they can also contribute to decreasing the product’s carbon footprint. The next generation of biopolymers will be synthesized from non-edible and highly available plants and from agro-food and industrial wastes or by-products, which contribute to the progress of the circular bioeconomy [8]. Furthermore, some biopolymers are also biodegradable, and their subsequent articles can be disintegrable in controlled compost soil [9,10]. These materials comprise diverse types of carbohydrates, for instance, thermoplastic starch, cellulose and its derivatives, alginates, chitin or chitosan, pectin, animal-based proteins (e.g., silk, gelatin, or collagen), and plant-based proteins as well as lipids [1].

Amongst the biopolymer proteins, gelatin was the most frequently employed in active packaging. Gelatin is a natural water-soluble protein marked by odorless and the random configuration of polypeptide chains in aqueous solution [11]. It is acquired from the partial hydrolysis of collagen, a fibrous protein mainly found in certain parts of vertebrate and invertebrate animals as bones, skins, connective tissues, and tendons and its structure consists of rigid bar-like molecules that arranged in fibers inter-connected by covalent bonds [12,13]. The potential use of gelatin as a biopolymer could be attributed to the triple helix structure giving a remarkable physical strength; absorbing the UV radiation; and, therefore, slowing down the food chemical oxidation reactions [14,15,16]. Regarding gelatin’s functional properties, its water-binding capacity, film- and foam-forming ability, and emulsifying trend have made it an encouraging applicant for food packaging [17].

However, gelatin-based films have some limits including (i) poor elongation at break and tensile properties weaknesses, and (ii) when these films come in contact with moisture, they may swell or dissolve [18]. To reduce these drawbacks, gelatin was blended with various polymers such as lipids and/or polysaccharides. It was employed to enhance the extensibility, flexibility, elasticity, and mechanical features. For instance, sodium alginate (NaAlg), a nontoxic, biodegradable, biocompatible, and cheap hydrocolloid, can develop stable films with notable traits, viz., resistance, flexibility, water solubility, and low permeability to O_2_ [19].

Alginates are isolated from the cell walls of brown algae (e.g., *Laminaria digitata* and *Ascophyllum nodosum*), where they are established in the form of sodium, calcium, and magnesium salts of alginic acid; they can also be synthesized by microorganisms [20,21]. Alginate is a linear, anionic, water-soluble polysaccharide, and its most beneficial property is attributable to its aptitude to react with polyvalent metal cations, particularly with calcium ions. This characteristic has led to the improvement of mechanical properties, barrier properties, cohesiveness, and rigidity [22,23]. By increasing the concentration of cations during the gelation of alginates, a dense structure with reduced porosity is shaped and a decrease in the water content or the gel permeability appears [22,24].

Nevertheless, the low biological capacities of gelatin-NaAlg-based film limited its applicability [18,25,26]. Consequently, numerous attempts have been made to integrate plant extracts into the gelatin-NaAlg film to improve their functional properties [18,27,28,29]. In this respect, plant extracts have admitted an increasing focus on their high amount of phenolic compounds [30,31,32]. Established films produced by integrating plant extracts into films interestingly led to changed optical, physicochemical, barrier properties, and antioxidant and antimicrobial activities in comparison with films fabricated of individual components [33,34].

Some attempts were made to incorporate and characterize biodegradable films established from multiple plant extracts. For instance, amaranthus leaf extract [26], *Ginkgo biloba* extract [25], grapefruit seed extract [35], *Nigella Sativa* [36], and *Opuntia Oligacantha* [37] were incorporated into films to improve their functional properties. Among the plant extracts with virtuous potential, beetroot (*Beta vulgaris* L.) peels have been considered to possess numerous biological activities (e.g., antimicrobial, antioxidant, anti-hypertensive, anti-inflammatory, anti-anxiety, anti-cancer, and anti-diabetic) [7,38,39]. With a low-cost source of bioactive compounds, beetroot peel was able to scavenge free and active radical species and antibacterial potential [40,41].

Worldwide human consumption of meat and meat products was nearly 346.14 million tons in 2018 [42]. Therefore, this consumption is expected to increase in the next years. Thus, this evidently leads to an increase in employing packaging films. In fact, several studies mentioned the application of active packaging films in beef [43,44] and turkey meat [45]. In addition, it is a promising alternative to conventional synthetic antioxidants in the meat industry by maintaining safety and quality; avoiding pathogenic bacteria proliferation; and, therefore, expanding the shelf life of food products [39,46]. To the best of our knowledge, no data are available that report on the impact of beetroot peels on meat conservation.

In this context, the present investigation studied the incorporation of beetroot peel extract (BPE) into the gelatin-NaAlg coating of raw minced beef meat. Firstly, the characterization of the mechanico-physical and optical characteristics and biological activities of the newly bioactive film were evaluated. In addition, here, we outlined the relationships linked to oxidative stability, microbiological and instrumental color measurements, and sensory properties.

## 2. Materials and Methods

### 2.1. Preparation of Beetroot Peels Extracts (BPE)

*Beta vulgaris* L. peels powder was prepared according to the method of Chaari et al. [40] by using water as solvent extraction. The mixture was incubated in a shaker (150 rpm) at 30 °C for 30 min and filtered then centrifuged (10 min, 10,000× *g*, 4 °C). The supernatants were collected and then evaporated in a rotary evaporator.

### 2.2. Betalains Quantification and Antibacterial Activity

The total betalains were quantified spectrophotometrically in terms of betacyanin (Bc) and betaxanthin (Bx) contents in BPE at 480 nm and 538 nm, respectively, according to the work of Righi Pessoa da Silva et al. [47]. Bc and Bx were expressed in mg/g of sample.

The minimum inhibitory concentration (MIC) values, representing the lowest concentration of BPE in which the microorganism did not depict visible growth after incubation, were determined against four foodborne bacteria: *Staphylococcus aureus* ATCC 6538, *Salmonella enterica* ATCC 14028, *Listeria moncytogenes* ATCC 19117, and *Escherichia coli* ATCC 8739, as described by Fourati et al. [48]. The test was performed in sterile 96-well microplates with a final volume of 100 μL per well. The corresponding concentrations of BPE were transferred to each successive well to obtain a twofold serial dilution of the original sample. In fact, each sample was dissolved to final concentrations of 0.3125, 0.625, 1.25, 2.5, 5, 10, 20, and 40 mg/mL, and then filtered through 0.22 μm pore-size black polycarbonate filters (Millipore). To each test well, 10 μL of cell suspension was added to the final inoculum concentration of 10^6^ CFU/mL of the bacterium. Plates were then incubated at 37 °C for 24 h. As an indicator of microorganism growth, 25 μL of thiazolyl blue tetrazolium bromide (MTT) indicator solution (0.5 mg/mL) dissolved in sterile water was added to the wells and incubated at 37 °C for 30 min. The colorless tetrazolium salt acts as an electron acceptor and was reduced to a red-colored formazan product by biologically active organisms. Where microbial growth was inhibited, the solution in the well remained clear after incubation with MTT. The determination of MIC values was carried out in triplicate.

### 2.3. HPLC Analysis

Extracted fractions were analyzed qualitatively with high-performance liquid chromatography (HPLC) to evaluate betalains (Bc and Bx). HPLC with a DAD (Agilent Technologies 1260 Infinity) Zorbax Analytical 4.6 × 150 mm XDB-C18 column was utilized. The separation gradient was performed with two solvents, water with 0.1% acetic acid and acetonitrile (solvents A and B, respectively). The gradient elution mode started with 3% B for 3 min, 50% B for 5 min, 100% B for 5 min, 100% B for 5 min, and 3% B for 3 min. The flow rate of 0.8 mL min−1, with an injection volume of 50 μL, operated at 30 °C. The pigments (Bc and Bx) were followed at wavelengths of 480 and 538 nm, respectively.

### 2.4. Preparation of Film

The films were performed according to the method of Dou et al. [18] with slight changes. The gelatin (4%, (*w*/*v*)) was added to three different concentrations of BPE: 1 × MIC, 2 × MIC, and 4 × MIC, which corresponded to 0.25, 0.5, and 1%, respectively, and to a control film sample without BPE. These mixtures were stirred at 150 rpm at 50 °C for 30 min. Sodium alginate (NaAlg) at 3% was mixed for 20 min at 45 °C. Then, as a plasticizer, the glycerol (20%, (*w*/*w*)) was poured into the formed solutions and blended for 15 min at 45 °C. Thereafter, the film-forming solutions were put into a Petri dish and dried at 45 °C. The films (control, 1-BPE, 2-BPE, and 4-BPE) were peeled off and stored at room temperature in desiccators for further analysis.

### 2.5. Film Characterization

#### 2.5.1. Mechanical Properties

Tensile testing was conducted through employing universal testing equipment (MTS Criterion) at ambient temperature at a crosshead speed of 20 mm/min through a load cell of 10 kN. The tensile mechanical tests were carried out on specimens cut by an injection machine. The specimens tested were in accordance with the kind of dumbbells standard specifications (test “ISO”) according to French standard NF T 51-034 and were subject to deformation at constant speed until the break. Elongation at break (EB%) was measured during the test achieved at the Tunisian Packaging Technical Centre (Packtec) according to Guo et al. [43].

#### 2.5.2. Physical Characterization

- Swelling index (SI)

As reported by Daei et al. [49], different film samples were cut into small portions (2 × 2 cm), weighted, and submerged in water for two minutes. The samples were weighted again (W2) after further soaking. The following equation determines the % of SI:

$$\mathrm{SI}\;\%=\frac{\left(W2-W1\right)}{W1}\times 100$$

where W1: initial mass of the films (g) and W2: final mass of the film (g).

- Water solubility (WS)

WS of the films was carried out according to Nouraddini et al. [50]. The weights of the 2 × 2 cm sections of the film samples were measured and then stirred in 30 mL of distilled water. The samples were weighed after drying at 105 °C for 24 h and removing the excess water. The following equation was used:

$$\;\mathrm{WS}\;\%=\frac{\left(\mathrm{Wf}-\mathrm{Wi}\right)}{\mathrm{Wf}}\times 100$$

where Wi and Wf: initial and final mass (g) of the film samples, respectively.

- Moisture content (MC)

MC was carried out by weighing the films before and after drying at 105 °C for 24–30 h [51]. MC was calculated as follows:

$$\;\mathrm{MC}\;\%=\frac{\left(\mathrm{Wi}-\mathrm{Wd}\right)}{\mathrm{Wi}}\times 100$$

where Wi and Wd: the mass of the initial and dried film (g), respectively.

- Biodegradability

Piñeros-Hernandez et al. [52] described the biodegradability test of films by using soil degradation. The film samples were dried at 60 °C. Then, all films were placed at a depth of 4 cm in a plastic box involving soil. Thereafter, 20 mL of water was added every two days. After 10 days, the film samples were washed with water and then dried at 60 °C. The biodegradability was calculated on the basis of the % of weight loss (WL):

$$\mathrm{WL}\;\%=\frac{\left(W1-W2\right)}{W1}\times 100$$

where W1: the initial film weight (g) and W: the dried film weight (g).

- Film instrumental color evaluation

Color values of developed films were measured by a colorimeter (Hunter Associates Laboratory, Reston, Virginia) using the CIELab color scale to evaluate the parameters L* (lightness/brightness), a* (redness/greenness), and b* (yellowness/blueness). Three measurements were taken for each film [37].

#### 2.5.3. Biological Characterization

- Antibacterial activity

The antibacterial properties of the different films were tested using the inhibition zone method against all 4 bacteria [53]. A portion of each film (14 mm diameter) was sterilized and then put on the surface of a solid bacterial culture (10^6^ CFU/mL). After the incubation period (24 h at 37 °C), the diameters of inhibition were measured.

- Total phenolic content and antioxidant activity

The total phenolic content (TPC) of the different film samples was quantified by the Folin–Ciocalteu method detailed by Liang and Wang [54]. Firstly, 0.5 mL of the film sample solutions was mixed with 2 mL Folin–Ciocalteu reagent. Then, 2.5 mL of 7.5% of sodium carbonate Na_2_CO_3_ solution was added. After 2 h, the absorbance was monitored at 765 nm. The results were stated as milligram gallic acid equivalent/g weight of the film (mg GAE/g film).

The antioxidant activity of the film samples was assessed by the method of Parveen et al. [55]. A 0.5 mL aliquot of sample solution was mixed with 0.5 mL 2,2-diphenyl-1-picryl hydrazyl 0.1 mM (DPPH). The reaction was carried out for 30 min at room temperature in the dark. The absorbances of the mixtures were quantified at 517 nm. This activity was calculated as follows:

$$\mathrm{DPPH}\;\%=\frac{\left(A\;\mathrm{control}-A\;\mathrm{sample}\right)}{A\;\mathrm{control}}\times 100$$

where A control and A sample are the measured absorbance of the control and sample, respectively.

### 2.6. Analysis of Beef Meat Samples

The raw beef (*Bos taurus*) meat was purchased from a local market (Sfax, Tunisia). Then, samples were minced (10 mm plate followed by 8 mm plate) by a meat grinder. They were placed in insulated polystyrene boxes on ice and transferred to the laboratory within 1 h of purchase. Concerning the conditioning of raw minced beef for storage at 4 °C studies, four equal portions (600 g of each) were placed separately in sterile plastic bags.

Meat samples were packed in the control, 1-BPE, 2-BPE, and 4-BPE film samples, which corresponded to 0, 0.25, 0.5, and 1%, respectively, and were kept stored at 4 °C for 0, 3, 7, 10, and 14 days. For each experiment, all runs were applied to a single batch of raw minced beef meat. Each formulation produced one mixture homogeneous which was vacuum-stuffed into plastic casings to produce three “replicates”.

#### 2.6.1. Microbiological Analysis

Ten grams of each sample with 90 mL sterilized peptone water was placed and homogenized in a stomacher. Then, decimal dilutions of the samples were prepared to be inoculated into the solid culture medium plate. The microbial counts were performed for (1) aerobic plate counts (APC) incubated at 30 °C for 48 h in plate count agar (PCA) [56], (2) psychrotrophic total counts (PTC) incubated at 7 °C for 10 days in PCA [57], and (3) *Enterobacteriaceae* counts (EC) incubated at 37 °C for 24 h in violet red bile glucose agar [58].

#### 2.6.2. Physiochemical Analysis

- pH analysis

pH was assessed for the homogeneous mixtures of meat with distilled water using a proportion of 1:10, *w*/*v* [59]. A 5 g portion of the sample was homogenized in 50 mL of distilled water (pH 7.00), and the mixture was filtered. The pH of the filtrate was measured using a pH-meter (pH210 Microprocessor pH Meter, HANNA instruments, Kehl am Rhein, Germany) at each sampling point.

- Evaluation of protein and lipid oxidation

Sulphydryl (SH) content was measured using the DTNB method (5,5′-dithiobis (2-nitrobenzoic acid)) proposed by Zhang et al. [60] and was expressed in mmol sulphydryl/g of protein.

Protein carbonyl content (CC) was assessed through following the protocol described by Mtibaa et al. [61]. Obtained results were reported in nmol carbonyl/mg of protein.

Metmyoglobin (MetMb) was quantified as described by Wang et al. [62]. In brief, 10 g of each meat sample was blended with 50 mL of phosphate buffer K_3_PO_4_ (pH 6.8, 0.04 M) followed by centrifugation (3000× *g*, 30 min). After filtration, the absorbance of the filtrates was valued at 582, 525, 557, and 503 nm. The % of MetMb was quantified using the formula provided by Wang et al. [62].

Regarding lipid oxidation, in our study, we evaluated peroxide values (PV), conjugated dienes (CD), and thiobarbituric acid reactive substances (TBARS).

PVs of meat samples were evaluated using the method of Folch detailed by Mtibaa et al. [61]. PV results were expressed in m equivalents of peroxide per kg of meat.

CDs were quantified according to the procedure of Mtibaa et al. [61], and the findings were reported as μmol/mg of meat sample.

Finally, TBARS values were carried out according to the work of Eymard et al. [63]. Meat samples’ absorbance was quantified spectrophotometrically. Findings were expressed as mg malonaldehyde (MDA) per kg of meat.

#### 2.6.3. Instrumental Color Evaluation and Sensory Evaluation

The surface color of the packaged raw minced beef meat was evaluated at 3 different points of each sample by measuring L*, a*, and b*. On the other hand, 30 laboratory panelists evaluated the sensory characteristics of raw minced beef samples. The evaluation of aroma, color, appearance and overall acceptability (OA) was performed on the basis of a hedonic scale rating from 1 (dislike extremely) to 9 (like extremely) [64]. It should be noted that all analyses were carried out in triplicate and periodically during 0, 3, 7, 10, and 14 days of refrigerated storage.

### 2.7. Statistical Analysis

Measurements were conducted during refrigerated storage on 0, 3, 7, 10, and 14 days and experiments with four treatments were utilized in a randomized complete block design. Moreover, at each storage time, three replications were performed. A two-way analysis of variance (ANOVA) was performed for all variables, as well as in cases of difference. The statistical significance of differences between mean values was analyzed using Tukey’s test at 5% significance using Statistical Package for the Social Sciences SPSS 19, (Ltd., Woking, UK).

To group samples on the basis of chemical oxidation, microbial counts, instrumental color, and sensory parameters during storage, all variables were auto-scaled prior to chemometrics application. By using XLSTAT software for Windows (v.2014.1.08, Addinsoft, New York, NY, USA), principal component analysis (PCA) and heat maps were performed to distinguish between samples at 0, 3, 7, 10, and 14 days (*n* = 20). The PCA type was Pearson (*n*), the biplot type was correlation biplot, and the coefficient was automatic. The Pearson correlation matrix and the PCA plots were performed. For heat maps, each cluster was determined by the Squared Euclidean distance matrix and the Ward method, generating dendrograms for all samples. Dendrograms were established to obtain a two-dimensional projection of the similarity or dissimilarity of the entire sample set.

## 3. Results and Discussion

### 3.1. Betalains Content

The Bc and Bx content in BPE were 32.17 ± 1.52 and 23.42 ± 1.09 mg/g, respectively, with 55.6 ± 2.68 mg/g of betalain pigment content collectively. The Bt content obtained in this study was higher than that reported by Vieira Teixeira da Silva et al. [65]. These authors studied the betalains of beetroot pulp extract, and the Bt level was 37 mg/g. It must be considered that the concentration of active chemicals in beetroot may vary depending on the variety, origin, or environment where the plant was harvested [66].

**Figure 1:**
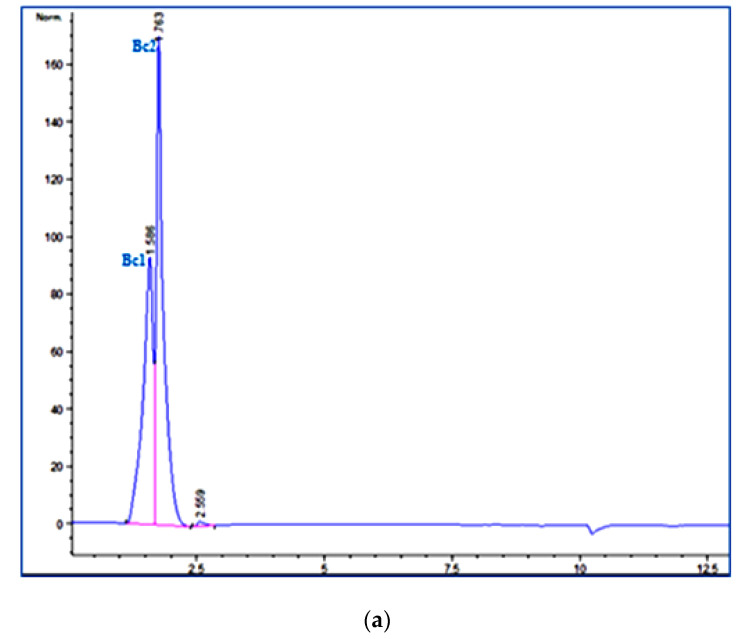

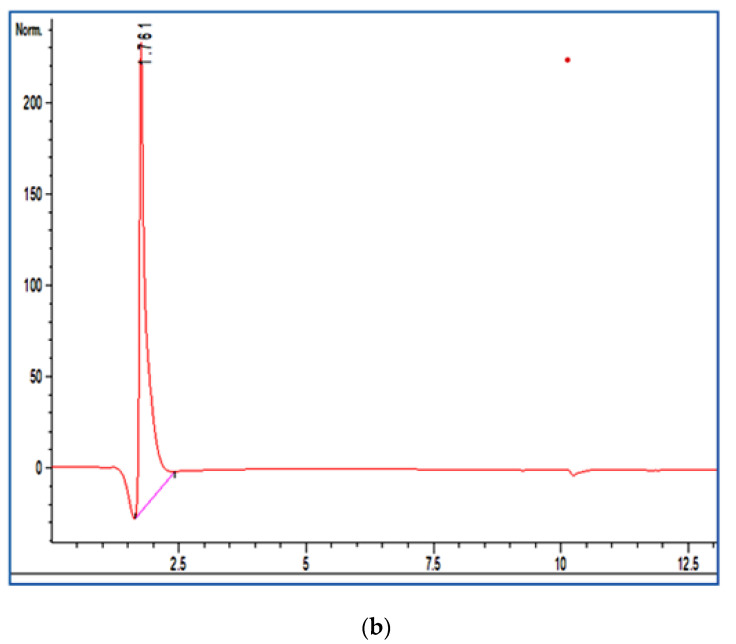
HPLC chromatograms corresponding to (**a**) Bcs (Bc1 and Bc2) and (**b**) Bx of the aqueous extract of beetroot peel (BPE).

The extracted fraction was analyzed with HPLC at 480 and 538 nm, which corresponded to the maximum absorption wavelengths of Bc and Bx, respectively. HPLC chromatograms showed the presence of two compounds (with a retention time of 1.597 (Bc1) and 1.711 min (Bc2)) at 480 nm and one at (with a retention time of 1.767 min) 538 nm, which suggested the presence of two Bcs (Figure 1a) and one Bx (Figure 1b).

### 3.2. Antibacterial Activity of BPE

BPE exhibited antibacterial activity against all studied foodborne pathogens. This extract showed the same MIC (MIC = 2.5 mg/mL) against Gram-positive bacteria *(S. aureus* and *E. coli*) and Gram-negative bacteria (*S. enterica* and *L. monocytogenes*). Thus, it was reported that BPE displays an antibacterial property against a wide range of microorganisms [40,67].

### 3.3. Film Properties

The BPE was blended into the packaging polymer solutions at three different concentrations, namely, 0.25, 0.5, and 1% equivalent to MIC, 2 × MIC, and 4 × MIC, respectively.

#### 3.3.1. Mechanical Characterization

TS and EB are two important characteristics of materials used for food packaging that affect the capacity of films. They reflect the mechanical strength and flexibility of the film [68]. Additionally, these parameters influence the integrity during storage, processing, and transportation of food products [69]. Mechanical properties of the newly developed films by BPE are presented in Table 1. When compared to the control film, adding BPE significantly reduced (*p* < 0.05) the TS. However, there was no significant difference (*p* > 0.05) between 1-BPE and 2-BPE films. The TS values ranged between 14.11 and 36.43 MPa. The maximum value of TS was reached by 4-BPE film, whereas the lowest TS was noticed for the control film samples. Comparable TS value (36.30 MPa) was obtained by Hu et al. [25]. These authors used 4% gelatin-based film with *Ginkgo biloba* ethanolic extract at 4%. Kanatt et al. [26] found a low TS value of 19.78 MPa when using gelatin-based film (2%) with Amaranthus leaf extract at 10%.

On the other hand, EB increased with BPE concentrations until reaching 91.1% when adding 4-BPE (Table 1). Similar trends were observed in carrageenan-gum-based films using beetroot extract [49]. Our results were higher than those obtained by Bitencourt et al. [70] (25.7%) when they tested the EB of the gelatin-based film (2% *w*/*v*) with Curcuma ethanolic extract. Moreover, the instant mechanical characteristics (TS, EB) were better than those found by Torres Vargas et al. [33]. These authors developed alginate-starch film with beetroot extract at 2 and 5%. According to Thakur et al. [71], mechanical characteristics of films may be improved by the interaction between gelatin, NaAlg, and polyphenols of plant extracts. In fact, polyphenols of natural extracts can create links with the polymer matrix’s constituents through covalent and non-covalent bindings [72,73].

#### 3.3.2. Physical Characterization

As reported in Table 2, the highest swelling percentage was exhibited by the control film (88.11%), followed by 1-BPE (67.53%), 2-BPE (60.86%), and 4-BPE film (56.06%). Likewise, with rising BPE concentrations, the films’ swelling significantly (*p* < 0.05) decreased. This finding has been confirmed by Aydin’s [74] study, who found that increasing of *Hibiscus sabdarifa* extract concentration provoked an SI decrease. Hence, the interaction of polyphenols of plant extract and carboxyl groups of alginate may have minimized the accessibility of these groups to water molecules [74].

Water solubility is a reflection of a film or coating’s water resistance [75]. When designing food packaging, it is crucial to consider the film’s water solubility [76]. The control film had the highest solubility % of all the generated films (100%). That was expected, especially considering the hydrophilic properties of gelatin and alginate [77]. It was observed that the solubility of the films is inversely proportional to the BPE concentrations used. The solubility drastically decreased after adding the BPE, as shown in Table 2. Furthermore, the lowest solubility was noted for 4-BPE (32.62%), followed by 2-BPE (40.80%) and 1-BPE films (55.70%). An almost similar value of solubility was presented by gelatin-based films (53.10%) with the addition of Amaranthus leaf extract [26]. Therefore, the solubility of these films tends to be enhanced with the increase in BPE concentration. The improved water solubility was related to the hydrophilic properties of betalains that can easily attach to water molecules [78].

The moisture content (MC) of all film samples is depicted in Table 2. Compared to the control film, the MC of 1-BPE, 2-BPE, and 4-BPE films were detected to be significantly low (*p* < 0.05). As mentioned in Table 2, the MC of the BPE films was remarkably reduced (*p* < 0.05) and ranged from 18 to 13%. These findings are in good agreement with those of Bitencourt et al. [70], who detected that gelatin-based films decreased the MC from 13 to 12% when the Curcuma extract was increased.

Regarding biodegradability, all the tested film samples were 100% biodegradable. These results enabled us to know that these films are biodegradable under environmental conditions. Due to its outstanding film-forming abilities and high biodegradability, gelatin has been frequently used [73]. In addition, Daei et al. [49] reported that the addition of beetroot extract could reduce the biodegradability time. Accordingly, the highest degradability was related to the film samples including the highest amount of beet extract [49]. Martucci and Ruseckaite [79] stated that a high rate of biodegradability (25%) of the gelatin-based film was found after 3 days.

#### 3.3.3. Optical Film’s Characterization

**Figure 2:**
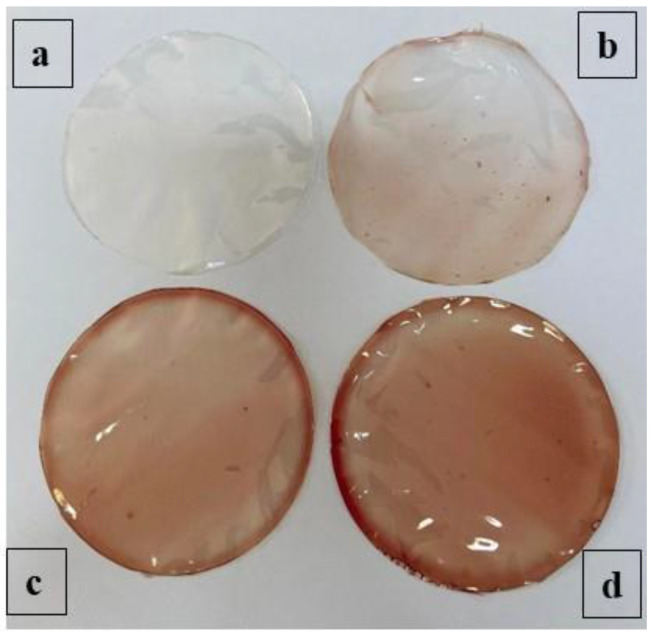
Visual aspects of (**a**) control (0%), (**b**) 1-BPE (0.25%), (**c**) 2-BPE (0.5%), and (**d**) 4-BPE (1%) films.

The color of films is a significant parameter that affects the package appearance and consumer acceptance of food products. The effects of BPE% on the instrumental color of films were elucidated in Figure 2.

In the same way, as mentioned in Table 3, the higher concentration of the BPE corresponded to lower L* values. In fact, L* values ranged from 47.92 to 42.35. The reduction of lightness as a consequence of the incorporation of beetroot extract into biopolymers has also been observed by other researchers [43,80]. Nevertheless, b* and a* values significantly increased (*p* < 0.05), and therefore, the BPE incorporation provided a reddish film color. Indeed, the increase in BPE concentrations augmented the intensity of the red color. This fact was confirmed by González et al. [81]—when beetroot was added (1.5%), the redness of the film was augmented.

#### 3.3.4. Biological Characterization of Developed Films

- Antibacterial activity

Table 4 displayed the antibacterial activity of all film samples. The control film did not exhibit any inhibitory effect on the tested bacteria. Furthermore, BPE films revealed considerable antibacterial activity, which progressively increased with the augmentation of BPE concentrations. It should be noted that this activity was more pronounced against *S. enterica* (25 mm) and *E. coli* (23 mm), which was exhibited by the 4-BPE film. These findings are in accordance with those reported by Hu et al. [25] who explored the antibacterial activity of *Ginkgo biloba* extract incorporated in gelatin film against *S. aureus* (13.6 mm). The antibacterial activity could be explained by the presence of high phenolic content in BPE films. BPE betalains are also well known to inhibit bacterial growth [40].

- Total phenolic content (TPC) and antioxidant activity (DPPH)

As stated in Table 5, with the increase in the concentration of BPE, TPC ranged from 21.85 to 23 mg GAE/mL, and the highest TPC was observed in the film with the highest level of BPE concentration (1%). The proportional increment of TPC and BPE could have been due to the high phenolic compounds present in the beetroot.

On the other hand, all the films displayed a high free radical scavenging ability (DPPH), except for the control film. As mentioned in Table 5, these films exhibited dose-dependent antioxidant activity. The percentage of this activity significantly increased (*p* < 0.05) with BPE concentration (from 30.32 to 70.68%). Moreover, some studies mentioned that the gelatin-based films formed with Amaranthus leaf extract or Ginkgo biloba exhibited, respectively, 42.58 and 24.70% of DPPH activity [25,26]. Our results indicate that gelatin-sodium alginate film containing BPE could be employed to prevent oxidation of packed foods.

### 3.4. Minced Beef Meat Analysis

#### 3.4.1. Microbiological Analysis

The maximum level of APC was detected in the control film, reaching 7.810 log CFU/g on day 10 (Table 6). APC growth was significantly (*p* < 0.05) reduced by the addition of BPE. APC of meat packed with 1-BPE, 2-BPE, and 4-BPE films were 6.018, 5.520, and 5.102 log CFU/g, respectively. According to Jridi et al. [82], meat samples coated with gelatin films and henna aqueous extract provided comparable APC on the eighth day (5.78 log CFU/mg). Additionally, the instant results showed that the APC decreased significantly with the increase in BPE concentrations (*p* < 0.05). Interestingly, until the 14th day of storage, the APC of meat packaged with 2-BPE and 4-BPE films did not reach 6.7 log CFU/g of meat [83].

Table 6 represents the microbial counts at each sampling time. The PTC increased considerably (*p* < 0.05), attaining higher counts in control samples. The films with BPE significantly reduced (*p* < 0.05) PTC in beef meat samples. In fact, at the end of storage, PTC of meat packaged with 1-BPE, 2-BPE, and 4-BPE films were respectively 6.98, 6.71, and 6.18 log CFU/g. Hence, these films allowed a prolonged shelf life for meat up to 14 days at 4 °C. Jridi et al. [82] revealed in their study that PTC of meat coated with gelatin and henna aqueous extract film was 5.62 log CFU/g after 8 days of storage.

Regarding Enterobacteriaceae, the counts were held under the threshold (2 log CFU/g) for meat packaged with BPE films during storage [83]. All meat samples packaged with BPE films showed low counts (*p* < 0.05) when compared with the control (Table 6).

Marrone et al. [46] pointed out the direct influence of beetroot extract on microbial growth in black Angus burger samples. These authors reported that the beetroot extract inhibited bacterial growth in beef meat samples. Thus, BPE films showed a remarkable antimicrobial dose-dependent effect against the tested bacteria. The impact of BPE films seemed to be related to dosage and storage duration. Lozano et al. [84] showed that beetroot films with a higher level of plant extract (5%) exhibited high antimicrobial activities against mesophilic aerobic bacteria, coliforms, and fungi. Indeed, the low bacterial count observed in raw meat packaged with BPE could be attributed to the phenolic and betalain compounds [85]. These compounds can destroy the membrane of microorganisms by raising cell wall permeability, which may result in cell destruction [40,86]. Hence, films could serve as supporters of antibacterial agents, which may be immobilized into the film matrix and then act when they are in contact with food products [87].

#### 3.4.2. Physiochemical Analysis

- pH analysis

During all days of storage, the pH values of the packed meat without BPE were noticed at the maximum level (Table 7). Low pH values were pointed out in the meat samples packaged with BPE films. These results are in line with a previous study conducted by Jridi et al. [82] on beef meat incorporated in active film with Lawsonia inermis extract (50 μg/mL). In addition, our results are in good agreement with the findings of Berizi et al. [88]. These authors investigated the combined effects of chitosan (Ch) and pomegranate peel (PPE) extract at 4% on the overall quality of rainbow trout during the frozen storage. At the end of storage, for the Ch + PPE at 4% samples, the pH was significantly lower compared to the control. In fact, pH values in the control and Ch + PPE4 groups were, respectively, 7.29 ± 0.36 and 6.72 ± 0.95. On the other hand, Langroodi et al. [44] used grape seed extract at 2% (GSE) as an active packaging agent to preserve turkey meat. These authors observed a significant decrease in pH values along the storage time (20 days) for analyzed batches. Xiong et al. [89] developed a chitosan-gelatin edible coating system incorporating grape seed extract at 5% and investigated its effect on the preservation of fresh pork during cold storage at 4 ℃ for 20 days. pH changes occurred from day 15 to day 20, and the pH values of the pork loin were significantly influenced (*p* < 0.05) by the grape seed extract in chitosan-gelatin edible coating treatments. At day 20, the pH values of all coated pork samples were much lower than the untreated samples, indicating that the pork was better preserved with the coating. Guan et al. [90] also suggested that the incorporation of extract at 0.1% sage extract, 0.1% oregano extract, and 0.01% grape seed extract (*w*/*v*), named SOG, affected the pH amount of the hairtail fish balls during 15 days at 4 °C. In this sense, the pH of the control increased from 6.87 to 7.33, compared to the pH of 6.84–6.96 of the SOG-treated sample.

Thereby, by extending the storage period, microorganisms developed more secondary metabolites and protein deamination, and by breaking down amino acids, ammonia was formed and accumulated in the meat, which ultimately raised the pH level [91,92]. Additionally, the generation of volatile amino acids by foodborne bacteria was among the main causes of the pH rise [91].

- Evaluation of protein oxidation

**Figure 3:**
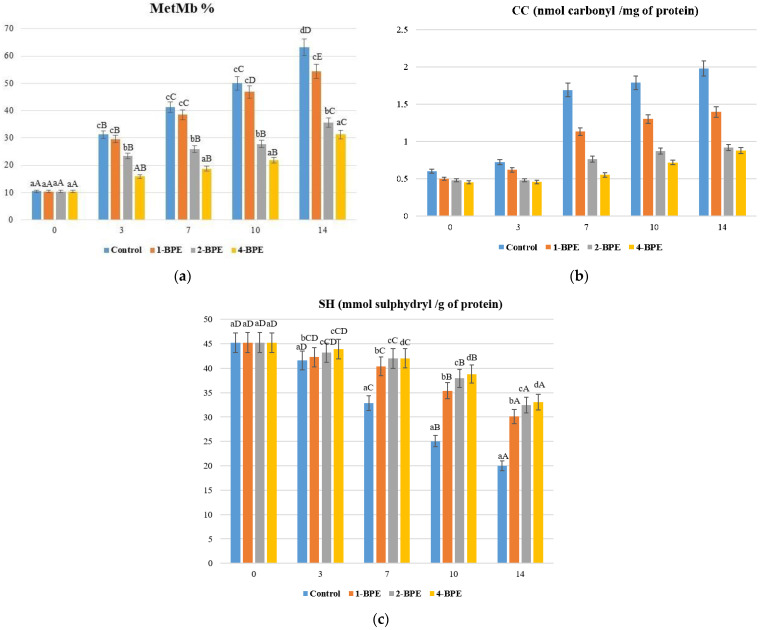
Effect of the films (control (0%), 1-BPE (0.25%), 2-BPE (0.5%), and 4-BPE (1%)) on (**a**) MetMb (%), (**b**) CC (nmol carbonyl/mg of protein), and (**c**) SH (mmol sulphydryl/g of protein) of raw minced meat beef stored at 4 °C for 14 days; ±: standard deviation (SD) of three replicates; a–d: mean values within all the samples not followed by a similar letter in the same column varied significantly (*p* < 0.05); A–E: mean values during storage not followed by a similar letter in the same line varied significantly (*p* < 0.05).

For all samples, the level of SH was reduced during storage, implying the formation of disulphide linkages (Figure 3a). After 14 days of storage, the minimum SH concentrations were detected in the control sample. On the contrary, for 4-BPE film samples, these values ranged between 45.23 and 33.05 nmol sulfhydryl/mg protein.

Protein carbonyls were formed from non-enzymatic and irreversible oxidative reactions of proteins, mainly by the deamination of the alkaline amino acids (lysine, arginine, and proline) [93]. As illustrated in Figure 3b, a significant (*p* < 0.05) increase in CC was detected during the storage of all meat samples. Recent findings were found to be similar to our study [94,95]. Low CC values of meat were perceived when the packaging samples formed with natural extracts, such as clove (*Syzygium aromaticum*), cinnamon (*Cinnamomum cassia*), and *Terminalia arjuna* extract.

Concerning MetMb values, all values were initially comparable (*p* > 0.05). Over 7 days, MetMb% increased significantly (*p* < 0.05) and rapidly, reaching 41.20% in the control sample, 38.45% in the 1-BPE sample, 25.87% in the 2-BPE sample, and 18.73% in the 4-BPE sample (Figure 3c). After the seventh day, the disappearance of redness was owing to the oxidation of oxymyoglobin to MetMb. Due to the antioxidant property of BPE, this process was retarded, leading to longer maintenance of meat redness.

Consequently, BPE film samples, especially 4-BPE film, served as a barrier to oxygen in the environment. The 4-BPE film could indirectly deplete oxygen occurrence during storage. Indeed, the efficiency of 4-BPE can be accorded to the extract’s phenolic compounds (mainly betalains) that exhibit metal chelating activity (chelating Fe^3+^ to Fe^2+^) [85], offering a high degree of protection against protein oxidation.

- Evaluation of lipid oxidation

**Figure 4:**
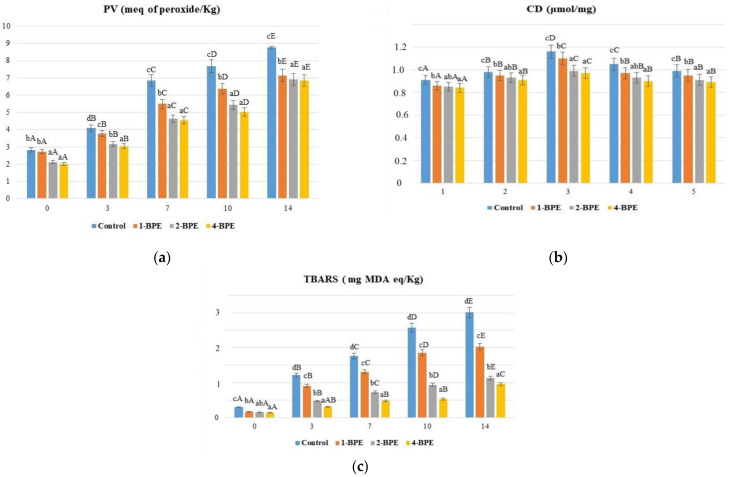
Effect of the films (control (0%), 1-BPE (0.25%), 2-BPE (0.5%), and 4-BPE (1%)) on (**a**) PV (meq of peroxide/Kg), (**b**) CD (μmol/mg of meat), and (**c**) TBARS (mg MDA-eq/Kg) of raw minced meat beef stored at 4 °C over 14 days; ±: standard deviation (SD) of three replicates; a–d: mean values within all the samples not followed by a similar letter in the same column varied significantly (*p* < 0.05); A–E: mean values during storage not followed by a similar letter in the same line varied significantly (*p* < 0.05).

Lipid oxidation is the major feature that affects the sensory qualities and nutritional value of meat and meat product quality [96]. This oxidation combines several reactions of poly-unsaturated fatty acids with oxygen by a free radical mechanism alongside the formation of many oxidation products, i.e., aldehydes, ketones, alcohols, acids, and hydrocarbons [97]. Regarding PV, Figure 4a depicted that along with storage time, PV increased (*p* < 0.05) significantly in all samples. On the 14th day, the meat sample fortified with 1% of BPE (4-BEP) revealed the lowest PV content (6.85 meq O_2_ /kg), followed by 2-BPE (6.92 meq O_2_ /kg) and 1-BPE (7.15 meq O_2_ /kg). Overall, the PVs of all the samples were less than 25 meq O_2_/kg, which is the accepted upper limit of fatty foods [98]. A decrease in PV in these samples is linked to hydroperoxide degradation [99].

CDs are products of the first level of lipid oxidation. As shown in Figure 4b, there was no noticeable difference (*p* > 0.05) in CD values at day 0. The decrease in CD level after the seventh day of storage could be interpreted by the development of secondary lipid oxidation products, e.g., aldehydes and ketones [97]. Interestingly, low values were noted in the samples of 1-BPE, 2-BPE, and 4-BPE films. A similar trend of CD was observed by Santos et al. [100] during beef patty storage at −18 °C using edible active film based on gelatin and *Malpighia emarginata* waste extract. BPE at 1% was more potent to delay the development of CD ranging from 0.84 to 0.97% over 7 days.

The beef meat samples were also valued for TBARS rates. These substances are employed as a crucial indicator for the assessment of oxidation’s secondary products such as aldehydes [101]. They occurred especially as a result of lipid oxidation of poly-unsaturated fatty acids [101]. As outlined in Figure 4c, TBARS rates increased significantly (*p* < 0.05) in all samples during storage. It is substantial to highlight that the activity of microbial pro-oxidant and endogenous enzymes and the liberation of heme iron from myoglobin are expected to cause a large rise in TBARS rates over storage time [97]. At day 0, similar TBARS rates were found (*p* > 0.05) in all meat samples. However, at the end of the storage, TBARS rates in the meat packaged with the film were higher than the limit level (3.01 mg MDA eq/Kg of meat). Conversely, the rates of TBARS were only 1.13 and 0.96 mg MDA eq/Kg of meat packaged with 2-BPE and 4-BPE films, respectively, which was inferior to the acceptable level of TBARS (2 mg MDA eq/Kg of meat) [102]. Notably, TBARS rates decreased significantly with the increase in BPE concentrations in the films (*p* < 0.05) compared to the control sample. This was in agreement with the study of Contini et al. [45], who stated that packaging carried out with citrus extract decreased TBARS rates in turkey meat for 4 days. Additionally, several studies corroborated the similar behavior of TBARS. These authors noticed the lowest TBARS values for refrigerated meat samples covered with gelatin-based films and henna (*Lawsonia inermis*) or *Malpighia emarginata* or garlic (*Allium sativum*) peel extracts when compared to their respective controls.

In view of these findings, it was possible to notice that 1% BPE incorporated in the gelatin was the most effective concentration to delay the development of PV, CD, and TBARS values during storage. Therefore, 4-BPE (1%) may have reduced the oxidation of beef meat by amplifying the activity of free radicals.

#### 3.4.3. Instrumental Color

With increasing time, the lightness values (L*) decreased for all samples. In fact, the use of the BPE at any concentration significantly (*p* < 0.05) decreased L* values. The decrease was highly found in meat samples packed with 4-BPE film (Table 8).

Regarding a*, the redness in all minced meat samples decreased during storage, and on the 14th day, the meat sample coated with control film significantly exhibited the lowest a* value (9.56) as compared to others (Table 8). All meat samples coated with BPE films had higher a* values (*p* < 0.05) than the control film samples. In fact, adding BPE decreased the L* values of the meat, while a* values raised and preserved the red color of the meat during storage. Therefore, the synthesis of MetMb through oxidation processes may be responsible for the decrease in a* values during the storage period [103]. Moreover, the behavior of betalains might be elucidated by their link to degradation by high temperature, light, and air [41].

As stated in Table 8, b* values increased significantly (*p* < 0.05) during storage. Packaged meat samples with BPE films showed the highest brownness level, since 1-BPE, 2-BPE, and 4-BPE represented 1.47, 2.23, and 3.6 b* values, respectively. However, the meat coated with the control film presented the lowest b* value (−0.80). The enzymatic browning reaction of phenolic compounds is potentially responsible for the increment of b* values.

#### 3.4.4. Sensory Evaluation

In the present study, the hedonic scale was utilized to assess each sample, with a score of 7 or higher being regarded as satisfactory. The meat coated with the control film sample and low concentration of BPE (0.25%) had no remarkable impact on sensory properties, but the higher amount of BPE (0.5 and 1%) was able to maintain the sensory traits of minced beef meat. All sensory properties including color, aroma, appearance, and OA (Table 9) decreased significantly (*p* < 0.05) throughout the storage period. The significant reductions in the sensory characteristics (*p* < 0.05) of meat coated with control film sample may have been due to the increased oxidation and microbial development during storage.

**Figure 5:**
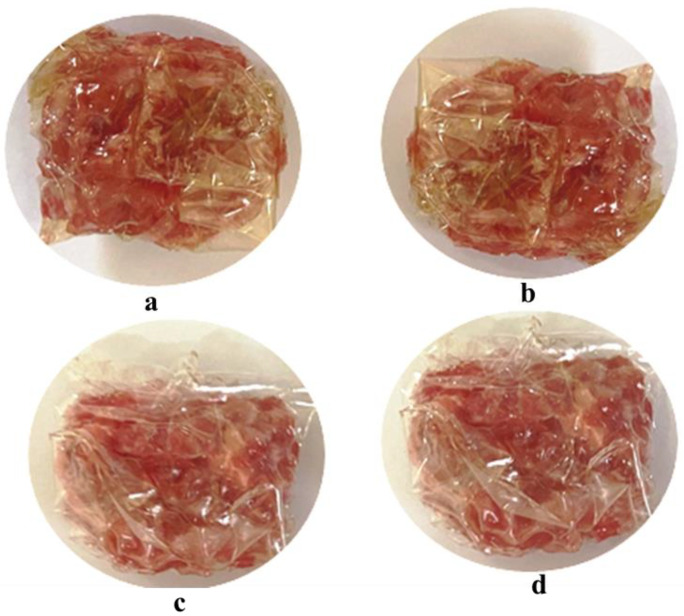
Visual aspects, after 14 days of storage at 4 °C, of minced beef meat packed with (**a**) control (0%), (**b**) 1-BPE (0.25%), (**c**) 2-BPE (0.5%), and (**d**) 4-BPE (1%) films.

The changes in the appearance of meat packaged with 2-BPE (0.5%) and 4-BPE (1%) films were not significant (*p* > 0.05) over 7 days, whereas a notable decline in color, aroma, appearance, and OA was detected in control samples (*p* < 0.05) over 14 days of storage (Figure 5a–d). It is clear that increasing BPE did lead to a relevant effect on sensorial properties (*p* < 0.05). Thus, 4-BPE film could considerably maintain these properties throughout storage time.

### 3.5. Chemometric Analysis

- Principal component analysis

**Figure 6:**
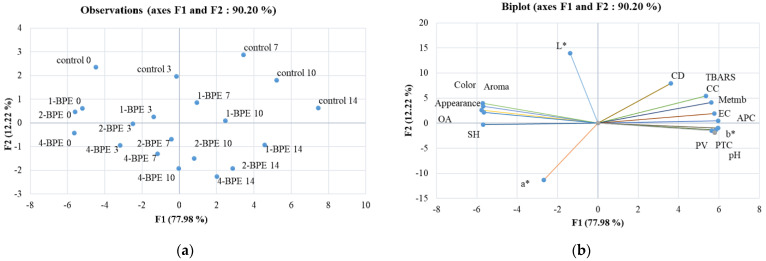
Loading plots of the two principal components (F1, F2) based on all samples (*n* = 20) (**a**) and on physicochemical, microbial growth, color, and sensorial property values of meat samples over 14 days of storage (**b**).

To obtain an extensive view of the impact of BPE films on minced beef meat’s instrumental color, lipid/protein oxidation, microbial growth, and sensory parameters, all data were subjected to the principal component analysis (PCA). Figure 6a,b depicts a biplot of the PCA loadings for two components (F1 and F2). F1 accurately predicted 77.98% of the variation, and F2 added an additional 12.22% of the study variation to its explanation.

Lipid and protein oxidation parameters (except SH), microbial counts, pH, and b* had a high positive loading on F1; these were opposed to sensory evaluation parameters, SH, L*, and a*, and they had negative loading on F1. Within the F2 component, the redness (a*) had a high negative loading; however, L* presented a strong positive loading.

- Heat maps

**Figure 7:**
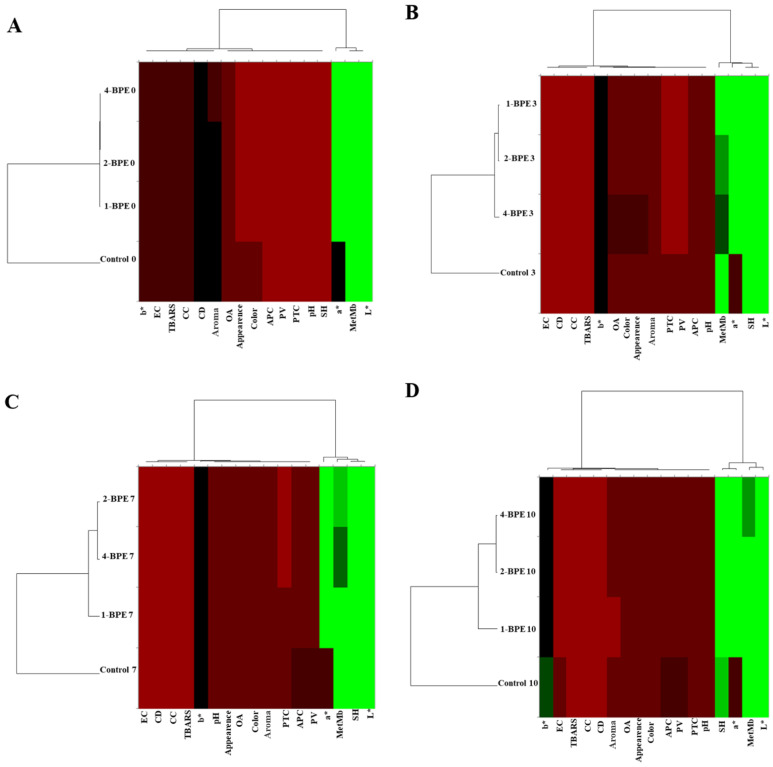

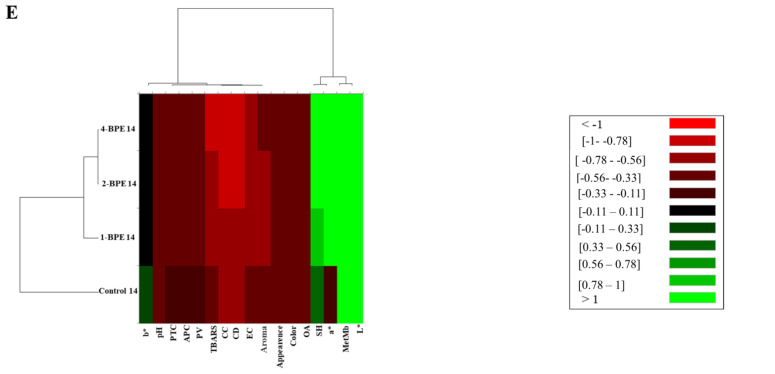
Heat maps of physicochemical, microbial growth, color, and sensorial property values of packaged raw meat samples on (**A**) day 0; (**B**) day 3; (**C**) day 7; (**D**) day 10; and (**E**) day 14 of storage.

For day 0, Figure 7A depicts the presence of two clusters, one of them regroups 1-BPE, 2-BPE, and 4-BPE that indicated the similarity between them. Conversely, on day 3, the dendrogram demonstrated the presence of three clusters (Figure 7B). In fact, MetMb correlated negatively with SH, a*, and L* values. A similar fact was depicted for the dendrogram on days 7, 10, and 14. This fact was affirmed by Wang et al. [104], who have shown that the stability of meat color and myoglobin autoxidation have a clear causal link. The oxidative reactions are the major reason for the loss of meat color, which is mainly related to the structural and chemical changes of myoglobin. Likewise, SH and color measurements’ correlation were in perfect agreement with the work of Estévez et al. [105], which highlighted the protective role of SH against oxidation and its impact on meat color quality. Additionally, Figure 7B depicts that on day 3, PV influenced sensorial properties, microbial growth, and pH. Smaoui et al. [106] affirmed that oxidative reactions have a substantial role in changing sensorial properties, especially the level of OA. In addition, according to some researchers, lipid oxidation of meat causes negative changes in sensorial properties, i.e., reducing consumer acceptability and off-aroma emission [99,107]. This fact could be related to the high susceptibility of the phospholipid fraction to oxidation [107]. Langroodi et al. [44] mentioned also that the sensory properties were linked to the microbiological investigations. For the reason of the high levels of microorganisms and lipid oxidation, the unsatisfactory sensory properties (off-aroma, off-colors, and an undesirable visual appearance) of the meat coated with control film samples scored lower [99]. Microbial spoilage, lipid oxidation, and a sensorial attributes relationship were perceived in camel meat incorporated into active packaging [99].

For days 3, 7, and 10, Figure 7B–D indicates the link between CC, CD, and TBARS values. This was approved by Zhao et al. [108], who showed a significant and positive correlation between CC and TBARS. These authors depicted that at the higher level of protein oxidation, high TBARS were noticed. That resulted in a high correlation found between lipid and protein oxidation [108]. The common agreement is that food-related oxidative processes including lipid oxidation and enzymatic reactions, which use oxygen as a catalyst, are connected to protein oxidation [97]. Additionally, high microbial loads and primary and secondary lipid oxidation, as well as protein oxidation parameters, were associated with all sensory attributes. In fact, all these characteristics were interrelated and implicated in meat quality. It is clear that chemometric instruments are widely used methods for evaluating the authenticity and quality of meat on the basis of its oxidative stability and color characteristics during storage. Numerous researchers have explored these methods [30,107,109].

## 4. Conclusions

Findings of this study found that the application of gelatin-based film enriched with BPE for packed raw minced beef meat resulted in the enhancement of quality features throughout refrigerated storage at 4 °C. Gelatin-NaAlg-based film including BPE has promising characteristics. In this line, the inclusion of BPE improved the mechanical, physical, and biological properties of the films. These properties were related to the amount of BPE incorporated. Enriched films with BPE at 1% revealed higher potency to delay protein and lipid oxidation, stabilize meat color, and decelerate microbial growth, therefore prolonging meat shelf-life. These effects are mainly caused by the antioxidants and antimicrobial compounds present in the BPE film. By expertly employing chemometrics technique, PCA, and heat maps, all data allow for helpful information to segregate all samples and connect oxidative and microbiological properties to sensory and instrumental color features using correlation models. The present investigation is a first attempt to evaluate the quality of minced beef meat treated with BPE by multivariate exploratory techniques. Consequently, the newly developed films may eventually be utilized for meat and meat product packaging due to being eco-friendly and economical alternatives to standard plastic films.

## Figures and Tables

**Table 1:** Mechanical characterization of films.

Sample	EB (%)	TS (MPa)
Control	61.21 ± 2.75 ^a^	14.11 ± 0.69 ^a^
1-BPE	66.10 ± 3.31 ^a^	23.20 ± 1.14 ^b^
2-BPE	70.10 ± 3.51 ^a^	25.03 ± 1.23 ^b^
4-BPE	91.10 ± 4.56 ^c^	36.43 ± 1.79 ^c^

Control (0%), 1-BPE (0.25%), 2-BPE (0.5%), and 4-BPE (1%); ±: standard deviation (SD) of three replicates; a–c: mean values within all the samples not followed by a similar letter in the same column varied significantly (*p* < 0.05).

**Table 2:** Physical characterization of films.

Samples	MC (%)	WS (%)	SI (%)
Control	19 ± 0.99 ^c^	100.00 ± 4.80 ^d^	88.11 ± 4.67 ^d^
1-BPE	18 ± 0.94 ^bc^	55.70 ± 2.73 ^c^	67.53 ± 3.58 ^c^
2-BPE	16 ± 0.83 ^b^	40.80 ± 2.00 ^b^	60.86 ± 3.23 ^b^
4-BPE	13 ± 0.68 ^a^	32.62 ± 1.60 ^a^	56.06 ± 2.97 ^a^

Control (0%), 1-BPE (0.25%), 2-BPE (0.5%), and 4-BPE (1%); ±: standard deviation (SD) of three replicates; a–d: mean values within all the samples not followed by a similar letter in the same column varied significantly (*p* < 0.05).

**Table 3:** Color parameters of films.

Samples	L*	a*	b*
Control	47.920 ± 2.16 ^c^	40.780 ± 0.03 ^a^	−0.80 ± 0.04 ^a^
1-BPE	44.327 ± 1.99 ^b^	43.537 ± 1.87 ^b^	1.47 ± 0.07 ^b^
2-BPE	46.757 ± 2.10 ^bc^	44.157 ± 1.90 ^bc^	2.23 ± 0.10 ^c^
4-BPE	42.350 ± 1.91 ^a^	45.052 ± 1.94 ^c^	3.60 ± 0.17 ^d^

L*: lightness; a*: redness and b*: yellowness. Control (0%), 1-BPE (0.25%), 2-BPE (0.5%), and 4-BPE (1%); ±: standard deviation (SD) of three replicates; a–d: mean values within all the samples not followed by a similar letter in the same column varied significantly (*p* < 0.05).

**Table 4:** Antibacterial activities of films.

Antibacterial Activity (mm)				
Sample	Anti-*S. aureus*	Anti-*L. monocytogenes*	Anti-*S. enterica*	Anti-*E. coli*
Control	0.00 ± 0.00 ^a^	0.00 ± 0.00 ^a^	0.00 ± 0.00 ^a^	0.00 ± 0.00 ^a^
1-BPE	15 ± 0.675 ^b^	18 ± 0.756 ^b^	19 ± 0.817 ^b^	16 ± 0.80 ^b^
2-BPE	16 ± 0.720 ^bc^	19 ± 0.798 ^bc^	21 ± 0.903 ^c^	21 ± 1.05 ^c^
4-BPE	18 ± 0.81 ^c^	20 ± 0.840 ^c^	25 ± 1.07 ^d^	23 ± 1.15 ^d^

Control (0%), 1-BPE (0.25%), 2-BPE (0.5%), and 4-BPE (1%); ±: standard deviation (SD) of three replicates; a–d: mean values within all the samples not followed by a similar letter in the same column varied significantly (*p* < 0.05).

**Table 5:** TPC and antioxidant activity of the films.

Samples	TPC (mg GAE/mL)	DPPH (%)
1-BPE	21.85 ± 0.98 ^a^	30.32 ± 1.42 ^c^
2-BPE	22.81 ± 1.03 ^a^	46.18 ± 2.17 ^b^
4-BPE	23.00 ± 1.04 ^a^	70.68 ± 3.32 ^a^

Control (0%), 1-BPE (0.25%), 2-BPE (0.5%), and 4-BPE (1%); ±: standard deviation (SD) of three replicates; a–c: mean values within all the samples not followed by a similar letter in the same column varied significantly (*p* < 0.05).

**Table 6:** Effect of films on the microbial load of APC, PTC and EC (log CFU/g) of raw minced meat samples stored at 4 °C for 14 days.

Days of Storage						
	Sample	0	3	7	10	14
APC	Control	2.08 ± 0.094 ^aA^	4.99 ± 0.224 ^bB^	6.789 ± 0.312 ^cC^	7.810 ± 0.366 ^dD^	8.990 ± 0.431 ^eE^
	1-BPE	2.05 ± 0.092 ^aA^	4.68 ± 0.210 ^bB^	5.432 ± 0.249 ^cC^	6.018 ± 0.282 ^dD^	7.242 ± 0.362 ^eE^
	2-BPE	2.02 ± 0.091 ^aA^	4.38 ± 0.197 ^bB^	5.221 ± 0.240 ^cC^	5.520 ± 0.270 ^dD^	6.530 ± 0.326 ^dD^
	4-BPE	2.01 ± 0.09 ^aA^	4.00 ± 0.180 ^bB^	4.980 ± 0.229 ^cC^	5.102 ± 0.239 ^cC^	6.172 ± 0.296 ^dD^
PTC	Control	2.36 ± 0.104 ^aA^	4.32 ± 0.194 ^cB^	5.96 ± 0.280 ^cC^	6.483 ± 0.311 ^cD^	7.50 ± 0.367 ^cE^
	1-BPE	2.35 ± 0.106 ^aA^	3.74 ± 0.168 ^bB^	4.23 ± 0.198 ^bC^	5.66 ± 0.272 ^bD^	6.98 ± 0.342 ^bE^
	2-BPE	2.35 ± 0.108 ^aA^	3.07 ± 0.138 ^aB^	3.57 ± 0.167 ^aB^	5.32 ± 0.255 ^abC^	6.71 ± 0.328 ^bD^
	4-BPE	2.34 ± 0.105 ^aA^	2.89 ± 0.130 ^aAB^	3.19 ± 0.149 ^aB^	5.06 ± 0.243 ^aC^	6.18 ± 0.302 ^aD^
EC	Control	<1	2.168 ± 0.101^dA^	3.147 ± 0.151 ^cB^	3.675 ± 0.180 ^cB^	4.161 ± 0.208 ^dC^
	1-BPE	<1	1.819 ± 0.085 ^cA^	2.149 ± 0.103 ^bB^	2.626 ± 0.128 ^cbB^	3.544 ± 0.177 ^cC^
	2-BPE	<1	1.439 ± 0.067 ^bA^	1.801 ± 0.086 ^aAB^	2.066 ± 0.101 ^aB^	2.884 ± 0.144 ^bC^
	4-BPE	<1	1.023 ± 0.048 ^aA^	1.715 ± 0.082 ^aB^	1.920 ± 0.094 ^aBC^	2.530 ± 0.102 ^aC^

Control (0%), 1-BPE (0.25%), 2-BPE (0.5%) and 4-BPE (1%); ±, standard deviation (SD) of three replicates; a–d: Mean values within all the samples not followed by a similar letter in the same column vary significantly (*p* < 0.05); A–E: Mean values during storage not followed by a similar letter in the same line vary significantly (*p* < 0.05).

**Table 7:** pH values of raw minced beef stored at 4 °C for 14 days.

Days of Storage					
Samples	0	3	7	10	14
Control	5.21 ± 0.234 ^aA^	5.46 ± 0.251 ^cB^	5.74 ± 0.270 ^dC^	5.88 ± 0.282 ^cC^	6.12 ± 0.300 ^cD^
1-BPE	5.20 ± 0.229 ^aA^	5.34 ± 0.246 ^bB^	5.52 ± 0.259 ^cC^	5.71 ± 0.274 ^bD^	5.94 ± 0.291 ^bE^
2-BPE	5.19 ± 0.236 ^aA^	5.27 ± 0.242 ^aB^	5.42 ± 0.255 ^bC^	5.68 ± 0.273 ^bD^	5.81 ± 0.285 ^aE^
4-BPE	5.19 ± 0.237 ^aA^	5.25 ± 0.247 ^aAB^	5.30 ± 0.249 ^aB^	5.61 ± 0.269 ^aC^	5.77 ± 0.283 ^aD^

Control (0%), 1-BPE (0.25%), 2-BPE (0.5%), and 4-BPE (1%); ±: standard deviation (SD) of three replicates; a–d: mean values within all the samples not followed by a similar letter in the same column varied significantly (*p* < 0.05); A–E: mean values during storage not followed by a similar letter in the same line varied significantly (*p* < 0.05).

**Table 8:** Color parameters (L*, a*, and b* ) of packaged raw minced meat samples stored at 4 °C for 14 days.

Days of Storage						
	Samples	0	3	7	10	14
L*	Control	46.213 ± 2.03 ^aC^	46.123 ± 2.12 ^bC^	45.750 ± 2.15 ^bBC^	45.200 ± 2.17 ^bB^	44.213 ± 2.17 ^bA^
	1-BPE	45.680 ± 2.01 ^aC^	45.390 ± 2.09 ^aC^	45.270 ± 2.13 ^bBC^	45.104 ± 2.16 ^bB^	44.100 ± 2.16 ^bA^
	2-BPE	45.406 ± 2.00 ^aB^	45.130 ± 2.08 ^aB^	44.106± 2.07 ^abAB^	43.400 ± 2.08 ^aA^	43.416 ± 2.18 ^abA^
	4-BPE	44.100 ± 1.94 ^aC^	43.780 ± 2.01 ^aB^	43.465 ± 2.04 ^aB^	43.115 ± 2.07 ^aAB^	42.955 ± 2.10 ^aA^
a*	Control	10.273 ± 0.45 ^aC^	9.155 ± 0.42 ^aB^	8.555 ± 0.41 ^aA^	8.820 ± 0.43 ^aA^	9.560 ± 0.47 ^aB^
	1-BPE	44.580 ± 1.96 ^bC^	43.420 ± 1.99 ^bBC^	42.550 ± 2.04 ^bB^	41.210 ± 2.01 ^bAB^	40.490 ± 2.02 ^bA^
	2-BPE	46.060 ± 2.02 ^bC^	44.780 ± 2.06 ^bB^	43.480 ± 2.08 ^bB^	41.796 ± 2.05 ^bA^	41.086 ± 2.05 ^bA^
	4-BPE	46.613 ± 2.05 ^bD^	45.940 ± 2.11 ^bC^	44.630 ± 2.14 ^bB^	42.050 ± 2.06 ^bA^	42.273 ± 2.11 ^bA^
b*	Control	11.470 ± 0.50 ^bA^	11.790 ± 0.54 ^aAB^	12.515 ± 0.59 ^bB^	14.115± 0.68 ^dC^	15.105 ± 0.74 ^cD^
	1-BPE	10.890 ± 0.48 ^abA^	11.560 ± 0.53 ^aB^	11.960 ± 0.56 ^abB^	13.000 ± 0.62 ^cC^	14.550 ± 0.71 ^bD^
	2-BPE	10.453 ± 0.46 ^aA^	11.276 ± 0.52 ^aB^	11.723 ± 0.55 ^aBC^	12.343 ± 0.59 ^bC^	14.253 ± 0.70 ^bD^
	4-BPE	10.136 ± 0.45 ^aA^	11.106 ± 0.51 ^aB^	11.576 ± 0.54 ^aBC^	11.700 ± 0.56 ^aC^	13.656 ± 0.67 ^aC^

Control (0%), 1-BPE (0.25%), 2-BPE (0.5%), and 4-BPE (1%); ±: standard deviation (SD) of three replicates; a–c: mean values within all the samples not followed by a similar letter in the same column varied significantly (*p* < 0.05); A–C: mean values during storage not followed by a similar letter in the same line varied significantly (*p* < 0.05).

**Table 9:** Effect of the films on appearance, color, aroma, and OA of raw minced meat samples stored at 4 °C for 14 days.

Days of Storage						
	Samples	0	3	7	10	14
Appearance	Control	8.2 ± 0.361 ^aE^	6 ± 0.270 ^aD^	5.10 ± 0.235 ^aC^	4.50 ± 0.212 ^aB^	3.62 ± 0.174 ^aA^
	1-BPE	8.5 ± 0.374 ^aD^	6.3 ± 0.284 ^bC^	5.35 ± 0.246 ^bB^	5.10 ± 0.240 ^bB^	4.40 ± 0.211 ^bA^
	2-BPE	8.25 ± 0.396 ^aD^	6.7 ± 0.302 ^cC^	5.70 ± 0.262 ^cB^	5.25 ± 0.247 ^bcB^	4.50 ± 0.216 ^bA^
	4-BPE	8.5 ± 0.396 ^aD^	6.87 ± 0.309 ^cC^	5.80 ± 0.267 ^cB^	5.35 ± 0.251 ^cA^	5.20 ± 0.250 ^cA^
Color	Control	8.45 ± 0.372 ^aE^	6.22 ± 0.280 ^aD^	5.30 ± 0.244 ^aC^	4.45 ± 0.209 ^aB^	3.50 ± 0.168 ^aA^
	1-BPE	8.5 ± 0.405 ^aD^	6.5 ± 0.293 ^bC^	5.33 ± 0.245 ^aB^	5 ± 0.235 ^bB^	4.40 ± 0.211 ^bA^
	2-BPE	8.33 ± 0.402 ^aD^	6.88 ± 0.310 ^cC^	6.35 ± 0.246 ^bB^	5.12 ± 0.241 ^bA^	5.01 ± 0.214 ^cA^
	4-BPE	8.5 ± 0.401 ^aD^	7.75 ± 0.349 ^dC^	6.45 ± 0.251 ^bC^	5.72 ± 0.245 ^cB^	5.10 ± 0.230 ^cA^
Aroma	Control	8.5 ± 0.374 ^aE^	6.01 ± 0.270 ^aD^	5.25 ± 0.242 ^aC^	4.31 ± 0.203 ^aB^	3.30 ± 0.158 ^aA^
	1-BPE	8.5 ± 0.383 ^aD^	6.20 ± 0.279 ^abC^	5.35 ± 0.246 ^aB^	5.25 ± 0.247 ^bB^	4.27 ± 0.205 ^bA^
	2-BPE	8.5 ± 0.378 ^aD^	6.34 ± 0.285 ^bC^	5.71 ± 0.149 ^bB^	5.30 ± 0.249 ^bAB^	5.00 ± 0.207 ^cA^
	4-BPE	8.5 ± 0.380 ^aD^	6.57 ± 0.296 ^cC^	6.01 ± 0.255 ^cB^	5.73 ± 0.251 ^cA^	5.40 ± 0.211 ^dA^
OA	Control	8 ± 0.356 ^aD^	5 ± 0.225 ^aC^	5 ± 0.230 ^aC^	4.71 ± 0.221 ^aB^	3.60 ± 0.173 ^aA^
	1-BPE	8 ± 0.358 ^aD^	6 ± 0.270 ^bC^	5.11 ± 0.235 ^abB^	5 ± 0.235 ^abB^	4.57 ± 0.219 ^aA^
	2-BPE	8.5 ± 0.382 ^aD^	6.75 ± 0.304 ^cC^	5.21 ± 0.240 ^bB^	5.11 ± 0.240 ^bAB^	5.00 ± 0.226 ^bA^
	4-BPE	8.5 ± 0.374 ^aE^	7.25 ± 0.315 ^dD^	5.65 ± 0.260 ^cB^	5.50 ± 0.248 ^cAB^	5.25 ± 0.230 ^cA^

Control (0%), 1-BPE (0.25%), 2-BPE (0.5%) and 4-BPE (1%); ±, standard deviation (SD) of three replicates; a–d: Mean values within all the samples not followed by a similar letter in the same column vary significantly (*p* < 0.05); A–E: Mean values during storage not followed by a similar letter in the same line vary significantly (*p* < 0.05).

## Data Availability

Data is contained within the article.
